# Environmental correlation and epidemiologic analysis of COVID-19 pandemic in ten regions in five continents

**DOI:** 10.1016/j.heliyon.2021.e06576

**Published:** 2021-03-27

**Authors:** Nadim Sharif, Mithun Kumar Sarkar, Shamsun Nahar Ahmed, Rabeya Nahar Ferdous, Nasir Uddin Nobel, Anowar Khasru Parvez, Ali Azam Talukder, Shuvra Kanti Dey

**Affiliations:** a Department of Microbiology, Jahangirnagar University, Savar, Dhaka 1342, Bangladesh; b Department of Microbiology, Bangladesh University of Health Sciences, Dhaka, Bangladesh

**Keywords:** COVID-19, Pandemic, Correlation, Temperature, Epidemiology

## Abstract

**Background:**

Coronavirus disease-2019 (COVID-19) has caused worldwide health emergencies during the last 6 months of 2020. Within very short time, severe acute respiratory coronavirus-2 (SARS-CoV-2) has infected over 64,516,333 people with 1,493,264 fatalities in 210 countries and regions. Previous studies have reported that environmental factors can affect the viability and transmission of SARS-CoV-2. This study aimed to determine the correlation of environmental factors with COVID-19 pandemic and epidemiology of COVID-19 across nine countries in five continents.

**Methods:**

Both environmental and health data were retrieved from various databases during January 1, 2020 to June 30, 2020. Mean value of environmental factors were calculated for weekly and daily cases and fatalities. Spearman correlation test was conducted.

**Results:**

In this study, most of the COVID-19 cases and fatalities were detected from regions (New York, Madrid, Lombardy, London and Sau Paulo) with 7 °C–25 °C mean temperature per day, 3 to 6 mean UV index per day and 14 km/h to 22 km/h mean wind velocity per day. Both cases and fatalities increased significantly after removing lockdown in Bangladesh, India, Brazil and South Africa. Over 50% COVID-19 patients were asymptomatic in every country except Brazil and Australia. Fever (>50%) was the most common symptom followed by cough (45%), tiredness (38%) and sore throat (30%), respectively. In India and Bangladesh over 70% of cases were reported in male. Significant correlation of COVID-19 cases with temperature and UV were detected in London, Lombardy, Madrid, New York and Dhaka.

**Conclusion:**

This is one of the first cross-country epidemiologic and correlation studies between environmental factors and COVID-19 pandemics. This study will help both local and international health organizations and policy makers to face the COVID-19 challenge.

## 1 Introduction

Severe acute respiratory coronavirus-2 (SARS-CoV-2), previously named as 2019-novel coronavirus (2019-nCoV) has caused the coronavirus disease-2019 (COVID-19) pandemic [1, 2, 3]. SARS-CoV-2 is from the family *Coronaviridae*. Members of this family namely, severe acute respiratory syndrome (SARS) and Middle East severe acute respiratory syndrome (MERS) had caused outbreak in the recent past. Highly infectious SARS, MERS and new SARS-CoV-2 cause infection of the respiratory system of human hosts [2, 3, 4]. First two isolates-human coronavirus 229E and human coronavirus OC43 from this family were detected in early 1960s in patients with common cold symptoms. Of note, serious infection of human respiratory system has been caused by other members- SARS-CoV (2003), HCoV NL63 (2004), HKU1 (2005), MERS-CoV (2012) and SARS-CoV-2 (2019) in the genera *Betacoronavirus* of this family [2, 3, 4]. However, none of the species were transmitted as fast as SARS-CoV-2 worldwide and none of them had caused pandemic except SARS-CoV-2 [5]. In late December 2019, first confirmed case of SARS-CoV-2 was reported from Wuhan, China. At present COVID-19 has become a major health issue around the world [4, 6, 7]. About 64,516,333 confirmed cases of COVID-19 have been reported from more than 210 countries and regions within eleven months. With a case fatality rate of 2.4%, COVID-19 has caused about 1,493,264 deaths worldwide during this time [8, 9].

SARS-CoV-2 is a positive sense virus with a non-segmented, single stranded RNA (ssRNA) genome of ~30,000 bases [3, 10]. The genome can act as direct mRNA as it contains 5′ cap structure along with a 3′ poly (A) tail. Among open reading frames (ORFs), 1a, 1b, 3a, 3b, 6, 7a, 7b, 8a, 8b and 9b are mostly reported from SARS-CoV-2 genome. First two ORFs- 1a and 1b occupy ~20,000 bases (two-third of genome) and encode for replicase proteins (non-structural proteins). About sixteen non-structural proteins (nsps) - nsp1 to nsp16 have been identified with defined functions. Later ORFs (~10,000 bases) encode for four major structural proteins-spike (S), envelope (E), membrane (M) and nucleocapsid (N). The genome order of coronavirus is 5′-leader-UTR-replicase-S-E-M-N-3′ UTR-poly (A) [3, 10, 11, 12].

Viruses associated with human respiratory tract infection have a trend to recur and transmit during winter than in other seasons [13]. However, seasonality of these viruses have not been well established [14, 15]. Environmental factors such as temperature, humidity, rain fall, UV intensity, wind velocity etc. are also related with seasonal changes [16]. Further, these environmental factors have definite roles in emergence and recurrences of infectious viruses. Emergence of the novel coronavirus (SARS-CoV-2) in Wuhan was also reported during winter in cold temperature [16, 17].

Possible mode of transmission of SARS-CoV-2 involves direct man to man contact, fomite and importantly via droplet nuclei [18, 19]. Above mentioned environmental factors have direct effect on the persistence and spread of SARS-CoV-2 through droplet nuclei. Of note, previous epidemiological and laboratory studies have reported that temperature is crucial for the survival and transmission of coronaviruses [16, 20, 21]. Temperature can directly affect the viability of coronaviruses in the environment and on the nonliving surfaces. Along with temperature, UV radiation has direct effect on the viability of coronaviruses in environment [16, 20, 21]. However, wind speed may have an important role for the spread of coronavirus in populated places. In fact, specifying the relationship of environmental factors like temperature, humidity, UV radiation, and wind speed with transmission, morbidity and mortality of SARS-CoV-2 will be vital to better understand the pandemic [16]. This study focuses to build baseline data by larger analysis of COVID-19 cases in ten cities/states in nine countries over five continents during January to May of 2020. This study will obviously provide a guideline to the international health organizations like WHO, CDC, NHS, policymakers and public by creating a better insight on the impact of environmental factors on COVID-19 pandemic.

Main aim of this study is to investigate the effect of environmental factors on transmission, morbidity and mortality of SARS-CoV-2 through droplet nuclei over five continents. Another aim of this study is to compare the case and fatality number in relation with environmental factors over different countries of five continents.

## 2 Materials and methods

### 2.1 Study area

This study focused on the coronavirus infection of five continents. Ten representative countries from five continents were studied based on the highest number of morbidity and mortality reports. China, Bangladesh, India in Asia; Spain, Italy, UK in Europe; USA in North America; Brazil in South America; South Africa in Africa; Australia in Oceania were selected by unbiased approach. Cities/States with the most number of cases and fatality were studied. This study included Dhaka (23.71°N to 90.41°E), Hubei (30.58°N to 114.27°E), Mumbai (19.07°N to 72.88°E) in Asia, Madrid (40.42°N to 3.7°W), Lombardy (45.46°N 9.19°E), London (51.51°N 0.13°W) in Europe, New York (40.71°N to 74.01°W) in North America, Sau Paulo (23.55°S to 46.63°W) in South America, Cape town (33.93°S to 18.42°E) in Africa and Sydney (33.87°S to 151.21°E) in Oceania. For most of the regions, the study period consisted of February 2020 to June 2020 except China. Each month was divided into four equal weeks (W1–W4) containing 7 days except the last week (W5) contained three days in January, one day in February, three days in March and two days in April and three days in May, respectively.

### 2.2 Data collection

Daily confirmed cases and fatality reports were collected from various online databases. For country wise case reports, WHO (www.who.int), Bing (www.bing.com/covid), worldometers (www.worldometers.info/coronavirus/) and Johns Hopkins University (https://coronavirus.jhu.edu/) website databases were analyzed. Country specific daily case reports and mortality reports of COVID-19 were retrieved from country specific websites. For Dhaka, data were collected from Institute of Epidemiology, Disease Control and Research (https://www.iedcr.gov.bd/website/) website, data for New York (https://www1.nyc.gov), Wisconsin (https://www.dhs.wisconsin.gov), Madrid (www.telemadrid.es), London (https://www.london.gov.uk/coronavirus/coronavirus-numbers-london), Sau Paulo (https://covid.saude.gov.br/), Sydney (https://www.health.gov.au), Cape Town (https://coronavirus.westerncape.gov.za/covid-19-dashboard) were collected from respective websites, data for Milan, Mumbai and Hubei were collected from WHO (www.who.int) website. Various environmental data for same period were collected from different databases including, meteoblue (www.meteoblue.com), AccuWeather (www.accuweather.com) and WeatherOnline (www.weatheronline.co.uk).

### 2.3 Statistical analysis

All of the data were analyzed using unbiased statistical approach. Case report and mortality reports were collected on daily basis. Sum of cases and fatalities were calculated weekly. Mean value for average ambient temperature, UV index, relative humidity and wind velocity were calculated weekly. Mean of average ambient temperature, number of daily cases and fatalities were analyzed in a two axis bar graph. Cross-country case number, fatality number within specific temperature range were analyzed. Spearman rank correlation test was conducted to detect the correlation between environmental factors and case number and fatality number [16].

## 3 Results

### 3.1 Descriptive analysis

Descriptive statistical analysis was conducted for the study regions. Descriptive analysis represents the actual burden of COVID-19 in different countries. In Africa, most of the cases were detected in South Africa's Western Cape with 61,172 cases and 1082 fatalities (during March 12, 2020 to June 30, 2020) having daily 15 °C mean temperature, 70% mean relative humidity, 7 mean UV index and 17 km/h wind velocity. In Asia, 1,24,067 cases and 1374 fatalities were calculated (during March 8, 2020 to June 30, 2020) from Dhaka, Bangladesh with daily 28 °C mean temperature, 70% mean relative humidity, 9 mean UV index and 12 km/h wind velocity. In Hubei, China, 67,990 cases and 3,280 fatalities were detected (during January 4, 2020 to June 30, 2020) with daily 10 °C mean temperature, 74% mean relative humidity, 6.2 mean UV index and 11 km/h wind velocity. In Maharashtra, India, 1,64,627 cases and 7,382 fatalities were detected (during March 10, 2020 to June 30, 2020) having daily 28 °C mean temperature, 65% mean relative humidity, 11 mean UV index and 11 km/h wind velocity.

Three cities with most number of cases and fatalities were analyzed in Europe. In Lombardy, Italy, 93,587 cases and 16,609 fatalities were detected (during February 21, 2020 to June 30, 2020) with daily 13 °C mean temperature, 70% mean relative humidity, 4 mean UV index and 9.5 km/h wind velocity. In Madrid, Spain, 74,669 cases and 8,217 fatalities were detected (during February 28, 2020 to June 30, 2020) with daily 15 °C mean temperature, 70% mean relative humidity, 5 mean UV index and 14 km/h wind velocity. In London, UK, 27,845 cases and 6,416 fatalities were detected (during February 12, 2020 to June 30, 2020) with daily 9 °C mean temperature, 75% mean relative humidity, 3.5 mean UV index and 20 km/h wind velocity.

In North America, New York with most number of cases and fatality was analyzed for the correlation. In New York, USA, 4,08,849 cases with 31,324 fatalities (during March 3, 2020 to June 30, 2020) with daily 9 °C mean temperature, 60% mean relative humidity, 5 mean UV index and 18 km/h wind velocity were specified. In Sau Paulo, Brazil, 2,48,126 cases with 14,030 fatalities were detected (during February 28, 2020 to June 30, 2020) with daily 20 °C mean temperature, 70% mean relative humidity, 11 mean UV index and 11 km/h wind velocity. In Sydney, Australia, Oceania, 3,076 cases and 49 fatalities were calculated (during March 7, 2020 to June 30, 2020) with daily 19 °C mean temperature, 71% mean relative humidity, 5 mean UV index and 15 km/h wind velocity.

### 3.2 Relationship of COVID-19 increase rate, fatality rate and detection rate with population density over time

**Figure 1:**
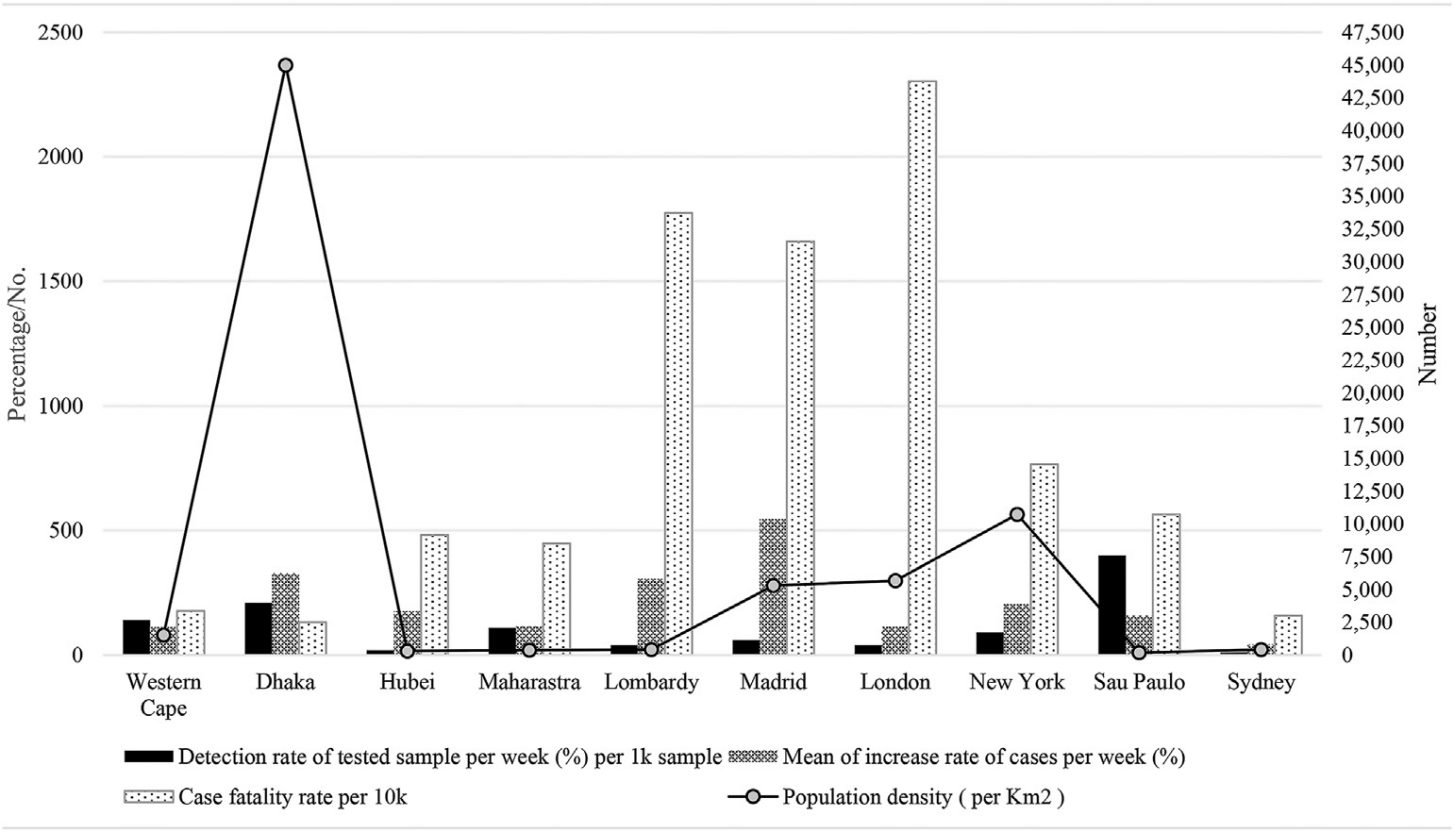
Relationship of COVID-19 increase rate, fatality rate and detection rate with population density in ten cities around the world.

The analysis was conducted by linear relationship with population density. Though Dhaka is one of the most populous cities in the world, the highest case fatality rate was detected in London. Dhaka was the most populous (45,000 person/km^2^) followed by New York (10,715 person/km^2^), London (5,666 person/km^2^), and Madrid (5,300 person/km^2^), respectively. Most number of COVID-19 confirmation tests had been performed in New York. However, detection rate of positive cases per 1000 samples per week was highest in Sao Paulo (400 cases), followed by Dhaka (210 cases), Western Cape (140 cases), Maharashtra (110 cases), respectively. Increase rate of COVID-19 cases over time was calculated. The highest mean value of increase rate of cases per week over the last six month was in Madrid (546% increase), followed by Dhaka (328% increase), Lombardy (305% increase), New York (204% increase), respectively. Case fatality rate was detected per 10,000 positive cases. The highest COVID-19 case fatality rate was detected in London (2,304), followed by Lombardy (1,775), Madrid (1,660) and New York (766), respectively (Figure 1).

### 3.3 Distribution of COVID-19 cases and fatalities in relation with temperature

**Figure 2:**
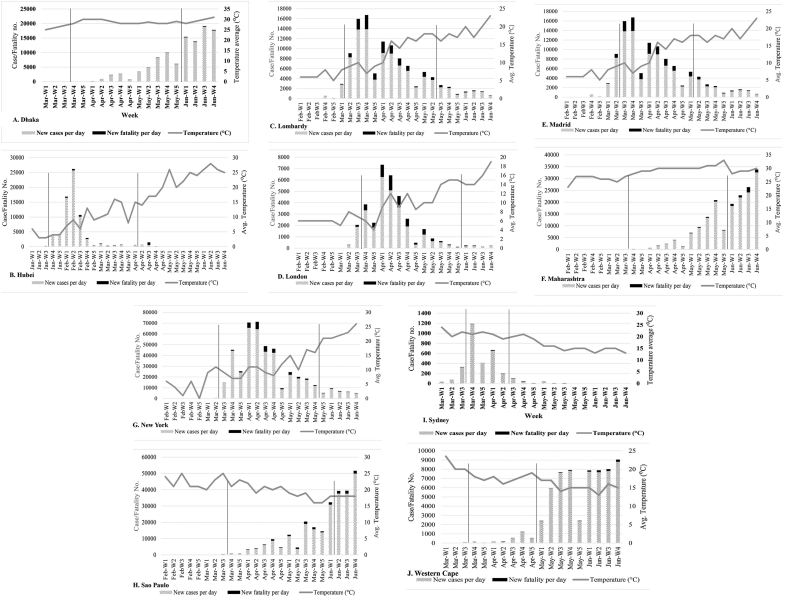
Weekly Distribution of COVID-19 cases and fatalities in relation with mean of average temperature per week in (A) Dhaka, (B) Hubei, (C) Lombardy, (D) London, (E) Madrid, (F) Maharashtra, (G) New York, (H) Sao Paulo, (I) Sydney, (J) Western Cape. The area inside two line indicates duration of lockdown.

Mean values of average ambient temperature were plotted against number of cases and fatalities in ten cities/states of five continents. Both the cases and fatalities associated with COVID-19 were higher in regions with lower temperature. About 90% cases and 85% mortalities in Dhaka were detected during May-W3 (day 8- day 14), 2020 to June-W4, 2020 having mean of average ambient temperature of 28 °C per day. The mean of average ambient temperature was 30 °C two weeks before the peak infection of COIVD-19 in Dhaka (Figure 2A). In Hubei, China 97% (65,872 of 67,990) cases and 70% (2,290 of 3,280) fatality were detected during Jan-W4, 2020 to Mar-W4, 2020 with mean of average ambient temperature 9.5 °C per day. Of note, the mean of average ambient temperature was 3 °C two weeks before the peak infection of COIVD-19 in Hubei (Figure 2B).

In Lombardy, Italy, 89% (83,205 of 93,587) cases and 92% (15,248 of cases and 16,609) fatality were detected at 11 °C mean of average temperature per day during Mar-W2, 2020 to May-W2 2020 (Figure 2C). In London, UK, about 85% (23,534 of 27,845) morbidity and 91% (5,817 of 6,416) mortality were specified at 8.6 °C mean of average temperature per day during Mar-W4, 2020 to Apr-W4, 2020 (Figure 2D). About 86% (64,347 of 74,669) morbidity and 85% (6,986 of 8,217) mortality in Madrid, Spain were detected at 11.9 °C mean of average temperature per day during Mar-W3, 2020 to Apr-W4, 2020 (Figure 2E).

During May-W1 to June-W4 2020, about 93% (1,52,614 of 1,64,627) cases and 87% (6,394 of 7,382) fatalities were detected in Maharashtra India with mean of average ambient temperature 30 °C per day (Figure 2F).

**Figure 3:**
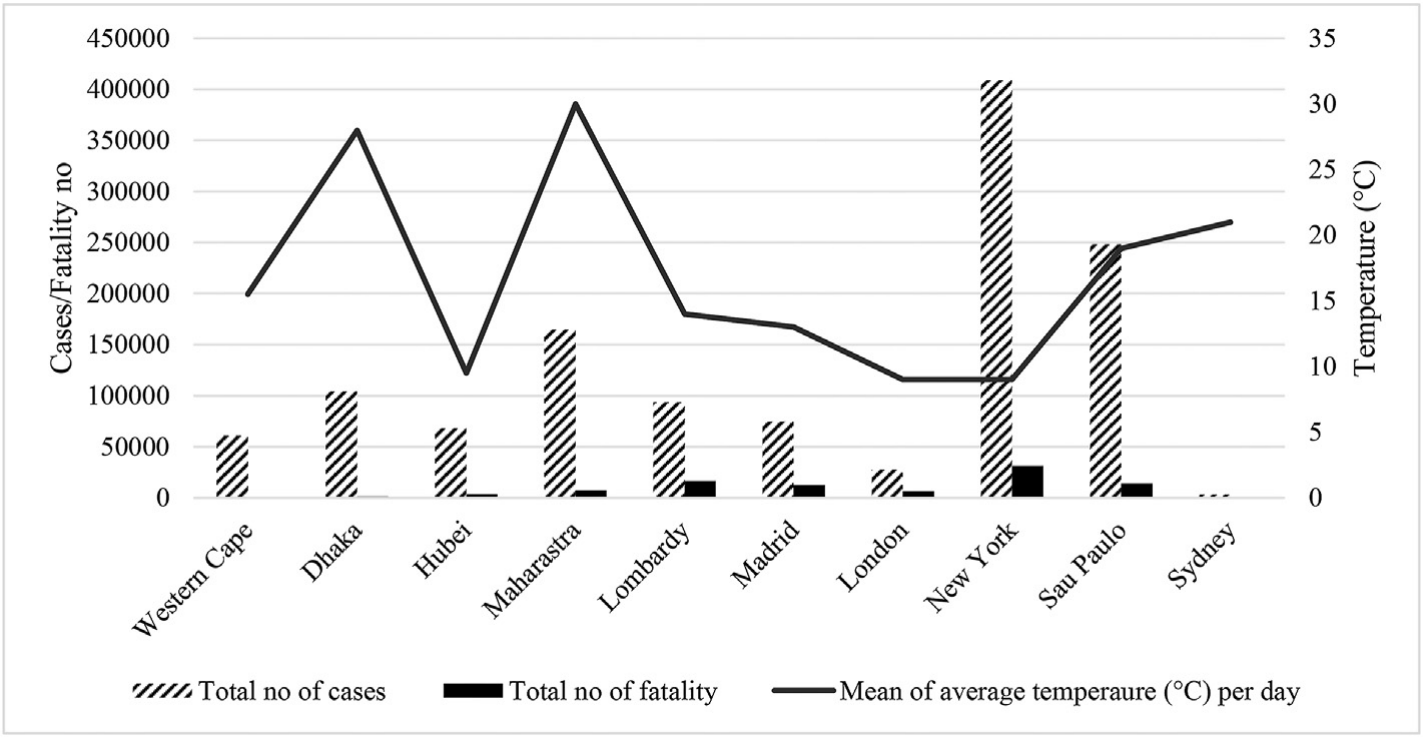
Relationship of total COVID-19 cases and fatalities with average temperature in ten different cities over five continents.

In New York, USA, about 96% (3,91,111 of 4,08,849) morbidity and 98% (30,652 of 31,324) mortality were detected at 9 °C mean of average temperature per day during Mar-W3, 2020 to June-W1, 2020 (Figure 2G) (Figure 3). In Sau Paulo, Brazil, 99% (2,45,787 of 2,48,126) cases and 97% (13,659 of 14,030) fatality were detected at 22 °C mean of average temperature per day during Apr-W2, 2020 to June-W4, 2020 (Figure 2H). In Sydney, Australia, 84% (2,575 of 3,076) cases and 37% (18 of 49) fatality were detected at 21 °C mean of average temperature per day during Mar-W1, 2020 to Apr-W1, 2020 (Figure 2I) (Figure 3.). Western Cape in Africa had most of the cases (98%, 59,821 of 61,172) and fatalities (98%, 1,060 of 1,082) during April-W4 to June-W4, 2020 with mean of average ambient temperature 18 °C per day (Figure 2J).

For spearman analysis, significant correlation were detected with the increase of cases and average temperature in Dhaka (0.211), Lombardy (0.466), London (.396), Madrid (0.374) and New York (0.322). Increased fatalities was also related with average temperature in Dhaka, Hubei, Lombardy, Madrid, London and New York (Table 1).

**Table 1:** Empirical values of Spearman correlation coefficient between environmental variables, number of cases and fatalities in ten cities over five continents during January to June, 2020.

Spearman correlation coefficient				Correlation analysis	
Wind speed	UV index	Relative humidity	Temperature average	Weather variables	
-0.136	0.254∗∗	-0.219	0.214	New cases	Western Cape
-0.224	0.223	-0.341	0.349∗∗	New fatality	
-0.165	0.128	0.132	0.211∗∗	New cases	Dhaka
-0.194	0.164	0.118	0.421∗	New fatality	
-0.214	0.198	-0.164	0.264	New cases	Hubei
-0.233	0.154	-0.211	0.382∗∗	New fatality	
-0.448∗	0.117	-0.175	0.177	New cases	Maharashtra
-0.136	0.239	-0.155	0.135	New fatality	
-0.235	0.243	0.339∗	0.466∗	New cases	Lombardy
-0.342∗∗	0.354∗∗	0.241∗	0.511∗	New fatality	
-0.293	0.295	-0.315∗	0.374∗	New cases	Madrid
-0.321	0.394∗∗	-0.142	0.411∗	New fatality	
-0.163	0.165	-0.119	0.396∗∗	New cases	London
-0.238	0.219	-0.247	0.416∗	New fatality	
0.139	0.387∗∗	-0.324∗	0.322∗∗	New cases	New York
0.229	0.347∗∗	-0.284	0.367∗∗	New fatality	
0.315∗	0.146	0.135	0.241	New cases	Sau Paulo
-0.112	0.144	0.219	0.164	New fatality	
-0.101	0.162	-0.143	0.184	New cases	Sydney
-0.154	0.198	-0.193	0.116	New fatality	

∗∗∗, ∗∗, ∗, 1%, 5% and 10% level of significance.

The effects of strict lockdown in COVID-19 pandemic was also analyzed. Case number increased significantly in Dhaka, Maharashtra, Sau Paulo and Western Cape after removal of strict lockdown. However, the COVID-19 cases and fatalities increased in London, Lombardy, New York and Madrid during lockdown (Figure 2).

### 3.4 Distribution of COVID-19 cases and fatality in relation with mean UV index

**Figure 4:**
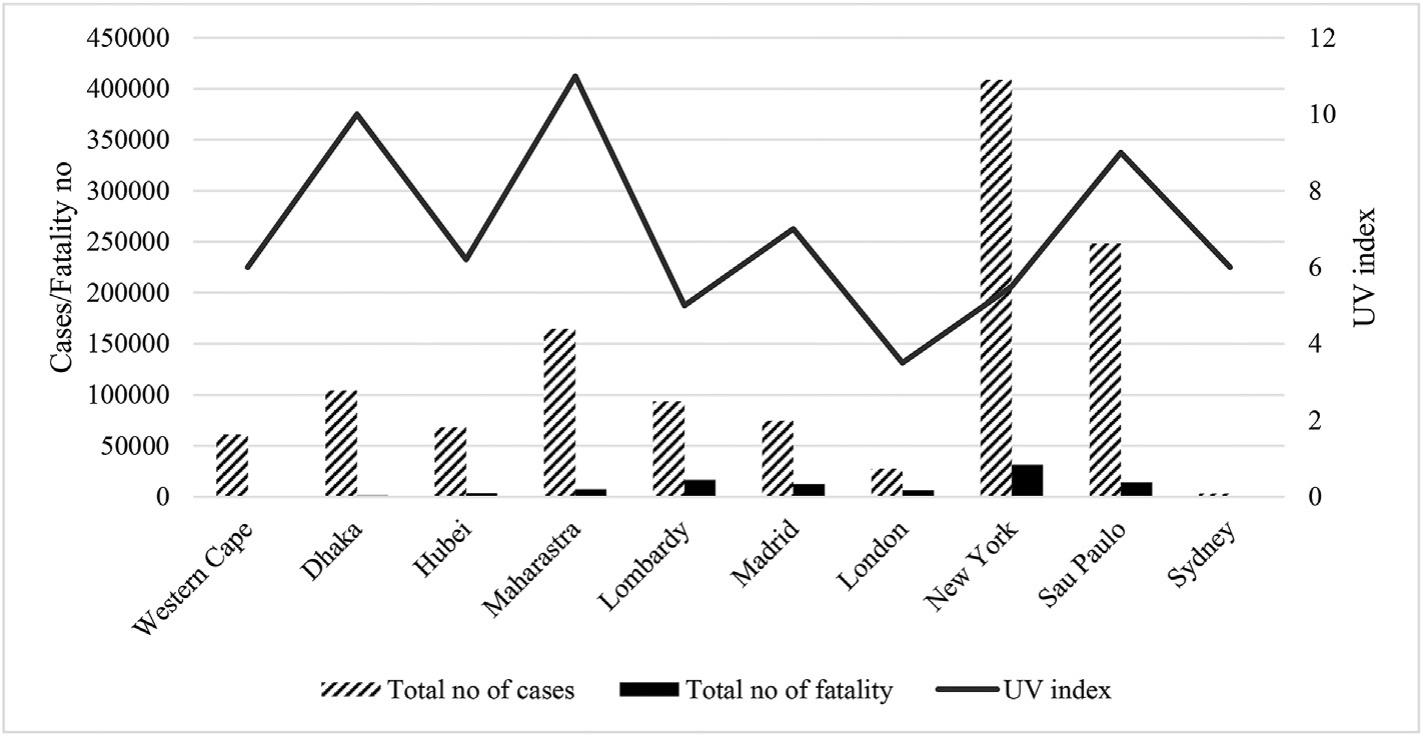
Relationship of total COVID-19 cases and fatalities with average UV index in ten different cities over five continents.

UV rays had direct killing effects on microbes. The severity of COVID-19 pandemic increased in regions with lower UV index. Mean UV index per day was calculated and plotted against case and fatality number. At 5.5 of mean UV per day 87% cases and 93% fatality was detected in Western Cape. About 90% cases and 74% mortality in Dhaka were detected at 9 mean UV per day. In Hubei, China 97% cases and 70% fatality were detected at 5.6 mean UV per day. However, about 98.5% cases and 98.6% fatality were detected in Maharashtra at 11 mean UV/day. In Milan, 87.5% cases and 91% fatality were detected at 4 mean UV per day. About 92% morbidity and 92.5% mortality were detected at 5 mean UV/day in Madrid. In London, 85.6% morbidity and 85.8% mortality were specified at 4 mean UV/day. In New York, 86% morbidity and 86.5% mortality were detected at 5 mean UV/day. About 94% cases and 95.8% fatality were detected at 8 mean UV/day in Sau Paulo. In Sydney, 84.5% cases and 40.9% fatality were detected at 5 UV/day (Figure 4). In spearman correlation analysis, significant correlation of COVID-19 fatalities were detected in Lombardy (0.354), Madrid (0.394) and New York (0.347).

### 3.5 Association of relative humidity and wind velocity with transmission of COVID-19

Relative humidity and wind speed play important role in the movement of droplet nuclei containing the virus and may have effects on the transmission of COVID-19. Average relative humidity during the study period varied from 65% to 80% per week in ten cities. Further, average wind speed per day varied from 10 to 20 km/h in the study regions. For the Spearman test, relative humidity was significant for new cases in Lombardy (0.339), Madrid (0.315) and New York (0.324) (Table 1.). Correlation between wind velocity and COVID-19 case number increase was detected in Maharashtra (-0.448) and Sau Paulo (0.315) (Table 1).

### 3.6 Clinical features of COVID-19 patients over five continents

Clinical symptoms of COVID-19 patients were analyzed in ten countries over five continents. Clinical features of COVID-19 varied greatly with severity and time globally. Three categories-asymptomatic (no symptom but positive), mild symptoms (having no serious complications), severe symptoms (leading to fatality over 70% of cases) were made for clinical feature analysis. Most of the COVID-19 patients are asymptomatic. The highest percentage of asymptomatic patients were detected in Bangladesh (80%), followed by England (78%), Spain (66%) and India (63%), respectively. Among mild symptoms, fever (over 50% cases) (measured >100.4°F [38 °C] or subjective), cough (over 45% cases), tiredness (over 40% cases), sore throat (over 38% cases), loss of appetite and smell (over 23% cases) were the most common symptoms over the five continents. In severe conditions over 70% of patients had breathing problem. Among other mild symptoms, diarrhea (≥3 loose stools in a 24-hour period), vomiting, myalgia, headache, runny nose, chill and rash were significant in COVID-19 patients (Table 2).

**Table 2:** Distribution of COVID-19 clinical symptoms in different regions during the first six months of the pandemic.

Country (cases)	Clinical features of COVID-19		
	Asymptomatic	Mild symptoms	Severe symptoms
Bangladesh (160000)	80%	**Fever,** dry cough, **tiredness**, aches, muscle pains, **sore throat**, diarrhea, conjunctivitis, **headache**, loss of taste or smell, rash, discoloration of fingers	**Shortness of breath,** chest pain
China (56000)	-	**Fever, dry cough**, **tiredness,** aches, muscle pains, **sputum production**, sore throat, headache, chill, nausea, vomiting, nasal congestion, diarrhea	**Shortness of breath, chest pain**
India (100000)	63%	**Cough, fever, sore throat,** headache, chill, nausea, vomiting, nasal congestion, diarrhea	**Shortness of breath**
England (4000000)	78%	**Cough, fever, loss of smell (anosmia)**, **tiredness,** headache, chill, nausea,	**Shortness of breath, chest pain**
Italy (33532)	40%	**Fever, cough,** chill, nausea, diarrhea	**Shortness of breath**
Spain (61075)	66%	**Fever,** chills, **tiredness, sore throat**, **cough,** headache or nausea, vomiting, diarrhea, **loss of smell**	**Shortness of breath**
Brazil (170000)	9%	Changes in taste and smell, **headache, fever**, **cough, palpitation**, vomiting, nausea, diarrhea	**Shortness of breath**
South Africa (150000)	50–60%	**Cough, fever, loss of smell,** headache, chill, nausea, vomiting, nasal congestion, diarrhea	**Shortness of breath**
USA (373,883)	40%	**Fever, dry cough, sore throat, myalgia,** loss of taste or smell, **headache,** chill, nausea, vomiting, nasal congestion, **diarrhea,** runny nose, abdominal pain	**Shortness of breath**
Australia (8000)	15%	**Fever, dry cough, sore throat,** myalgia, loss of taste or smell, headache,	**Shortness of breath**

The bold in the table indicated the most prevalent symptoms.

### 3.7 Distribution of COVID-19 cases by age and gender during January to June, 2020

**Figure 5:**
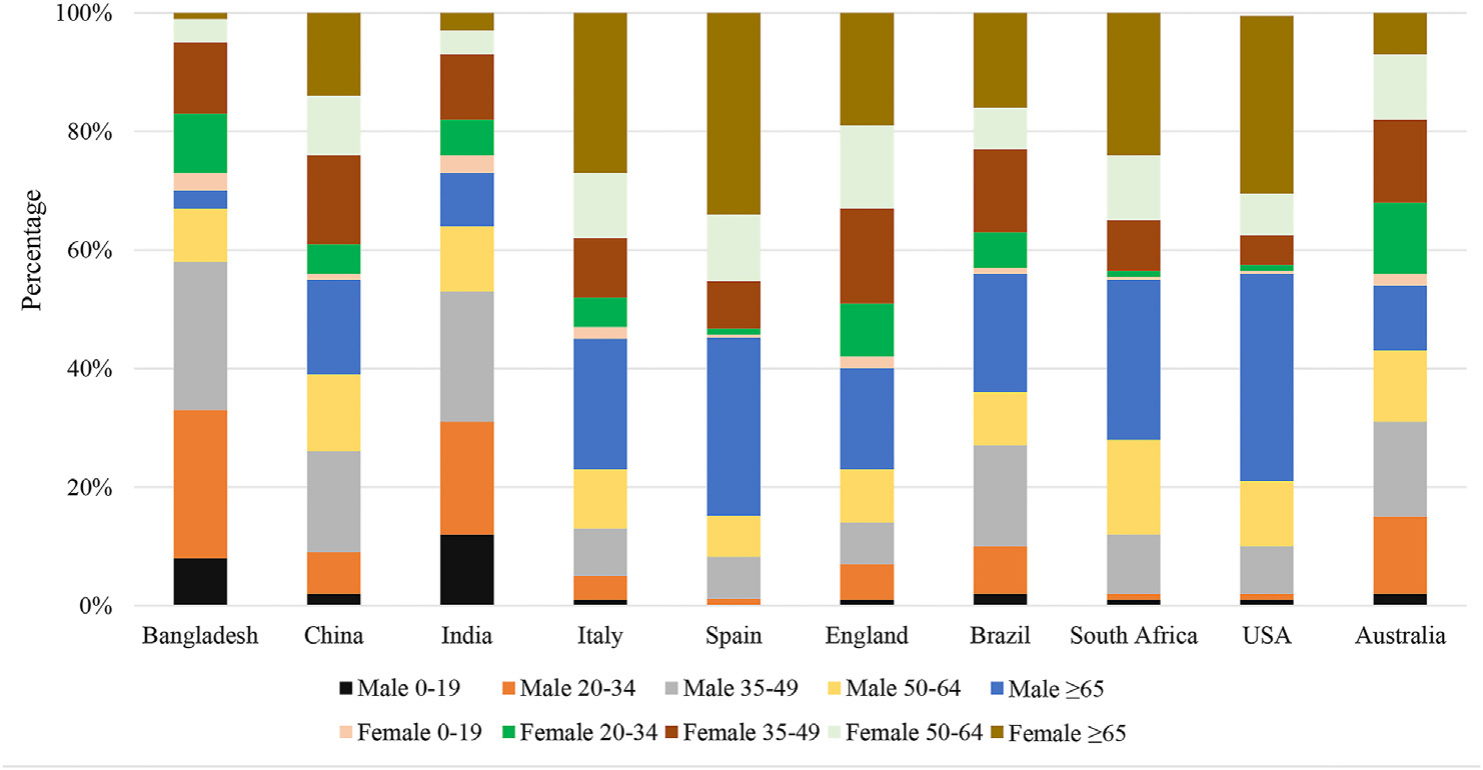
Distribution of COVID-19 cases in different age and gender groups in ten cities during January to June, 2020.

Both male and female patients were distributed in five age group [22]. High fatalities associated with COVID-19 were reported from over 55 years patients with comorbidity. In seven countries-Bangladesh, India, China, Brazil, South Africa, USA and Australia the percentage of male patients were greater than female. The highest difference in male to female ratio of COVID-19 cases was detected in India (2.4:1), followed by Bangladesh (2.3:1). The highest percentage of COVID-19 cases (34%) in Asia (Bangladesh, India, China) was detected in patients aged 25–39 years. However, in Europe (England, Italy, Spain), South America (Brazil) and North America (USA) the highest percentage (>50%) of cases were detected in patients aged ≥65. In Australia, the COVID-19 patients were distributed equally to every age group (Figure 5).

## 4 Discussion

Coronavirus disease-2019 (COVID-19) has become one of the major health burdens in 21^st^ century [23]. Though regional and environmental variation are present, severe acute respiratory coronavirus-2 (SARS-CoV-2) has been transmitted throughout the entire world within very short time [24]. Besides direct contact, SARS-CoV-2 is transmitted by droplet nuclei and nonliving objects. Therefore, different environmental factors can affect the viability and transmission of SARS-CoV-2 via droplet nuclei and nonliving objects [19, 24, 25]. This study focused to compare variation of COVID-19 morbidity and mortality in relation with environmental factors over ten different regions in ten countries across five continents.

Temperature is one of the major environmental factors that is related with seasonal and regional variation. Environmental temperature has strong and regulatory effect on the duration of survival and transmission of SARS-CoV-2 through droplet and nonliving objects. Several previous studies had reported regulatory effect of temperature on survival and transmission of other coronaviruses, SARS-CoV and MERS-CoV [26, 27, 28]. These studies reported optimum environmental temperature for SARS cases was between 16 °C to 28 °C. About 90% cases and fatalities in Hubei, Lombardy, Madrid, London, New York and Wisconsin were detected between 7.5 °C to 14 °C mean temperature per day. About 90% cases and fatalities were detected in Western Cape, Sau Paulo, Sydney, Dhaka and Maharashtra between 16.5 °C to 30 °C mean temperature per day. These findings were in good agreement with previous studies as higher frequency of the cases and fatality were detected below 20 °C around the world [16, 17]. However, case and fatality frequency and rate of increase of COVID-19 above 30 °C mean temperature per day are only a fraction of the frequency of cases and fatality detected below 30 °C. Of note, previous studies had reported reduced viability of SARS and MERS coronaviruses above 30 °C in the laboratory set up [29]. This study suggests that temperature is associated with the viability and rate of transmission of SARS-CoV-2. The spearman test suggests that average temperature and COVID-19 cases, average temperature and COVID-19 fatalities are strongly correlated worldwide. The findings are similar with previous reports of correlation from Hubei and New York [16, 17]. Of note, removal of strict lockdown caused significant increase of COVID-19 cases and fatalities in Bangladesh, India, Brazil and South Africa. However, the increase of temperature along with social distance measures played important role in reducing the COVID-19 cases in New York, London, Lombardy and Madrid. Further, the severity of COVID-19 has increased with the decrease of temperature in Asia, Europe, North America and South America. The onset of second wave was detected with the decrease of temperature globally.

Both the duration of lockdown and population density had significant influence on the COVID-19 pandemics. Removal of strict lockdown from populous countries has increased the cases and fatalities by many times. Of note, in Bangladesh, India, Brazil and South Africa the cases were under controlled during lockdown. However, in Europe and USA the cases reduced during the lockdown with increased temperature. These findings are in good agreement with previous studies in China, USA, Italy and Spain [30, 31].

In epidemiological analysis, we found the Asian countries- Bangladesh and India had significant difference in COVID-19 gender distribution. About 70% of the patients were male in Bangladesh which was also reported in India previously. However, in Italy, Spain, England, USA, Brazil, Australia and China the cases were distributed equally in male and female. In Bangladesh, India and China we found most of the cases in patients aged 35–49 years but in Italy, Spain, England, USA, South Africa and Brazil most of the cases were detected in patients aged >65 years. The proportion of young population is greater in Bangladesh and India. Further, the risk of infection is higher in young population in Bangladesh and India due to their vulnerable social and economic activities. Our findings on age and gender distribution of COVID-19 in nine countries are in similar with previous studies [2, 9, 11, 32, 33]. Clinical symptoms of COVID-19 varied with place and time. About 50% of the infected patients developed no symptoms in Bangladesh, India, Italy, Spain, England, New York, South Africa and Australia. Further, fever and cough were the most common symptoms in COVID-19 patients in the study regions. In serious cases, breathing problem was the most consistent symptom in patients of these ten cities. Clinical findings are also in strong agreement with previous studies in Italy, Spain, USA and England [2, 9, 11, 34, 35].

UV index is another major environmental factor that has antiviral effect. In several previous studies UV was found to be effective against SARS-CoV and MERS-CoV in laboratory set up [36, 37]. In the environment, the intensity and exposure time of UV index vary greatly that might be regulatory for the viability and transmission of SARS-CoV-2. Of note, majority of the cases and fatalities were detected at mean UV index 3 to 6 per day in the study regions. However, transmission of SARS-CoV-2 was detected at very high and extreme mean UV index of 9.3 and 11.7 per day in Dhaka and Maharashtra, respectively. For the Spearman test, this study reports that average UV index is significant for case and fatality number in eleven regions worldwide.

Relative humidity and wind speed also play important role for the seasonal and regional spread of coronaviruses [16, 17]. This study detected majority of cases and fatalities at 65%–80% mean relative humidity per day which is near optimum for droplet nuclei to survive longer in the environment. Highest mean wind speed 22 km/h per day was detected in New York followed by 17 km/h in Western Cape, 15 km/h in Sydney, 14 km/h in Lombardy, London, Madrid and 11 km/h in Dhaka, Hubei, Maharashtra and Sau Paulo, respectively. For the Spearman test, this study reports that average relative humidity and wind speed are significant for cases and fatalities in different regions worldwide. In some regions the association of relative humidity and COVID-19 cases were positive but in other regions the impact was negative. Further, the impact of wind speed was also positive in some regions and negative in other regions. These findings are similar to other previous studies in USA, China, UK and Spain [16, 17, 27]. Other environmental factors like, rain fall, snow fall, amount of precipitation were also analyzed against the number of COVID-19 morbidity and mortality. However, no strong correlation was detected among snow fall and amount of precipitation with COVID-19 pandemic. For the Spearman test, this study reports that average rain fall is significant for cases and fatalities of COVID-19. The impact of temperature on COVID-19 pandemic is significant. The incidence of COVID-19 changed greatly with seasonal variation. Both the first and second wave with increased cases and fatalities were reported in lower temperature. With the decrease of temperature in UK, USA, Italy, Spain, India, Bangladesh and China both the cases and fatalities started to rise up than before.

However, the study indicates a strong relationship between environmental factors and survival of novel coronavirus in the environment, several limitations should be considered. First, more environmental factors will be included to analyze correlation with case number and mortality. Second, more regions and countries should be included to interpret more accurate effects of environmental factors on SARS-CoV-2 survival and transmission.

## 5 Conclusions

To the best of our knowledge, this is one of the first cross-country comparative studies reporting correlation of environmental factors with COVID-19 transmission, morbidity and mortality over five continents. Our study finds significant level of correlation between environmental factors and SARS-CoV-2 survival. If direct contact is not avoided, social distance and personal hygiene are not maintained, environmental factors alone can't reduce the COVID-19 associated morbidity and mortality. Regions with mean temperature between 7 °C to 25 °C per day have most number of cases and fatality compared to regions with mean temperature above 25 °C per day. Unless direct contact is avoided and personal hygiene are strictly maintained, both the morbidity and mortality will increase in regions with warmer weather. This study claims that if necessary steps are not taken, COVID-19 pandemic will become worse in developed world during next winter with reduced temperature. These findings will provide guideline data to for policy makers and researchers to take necessary measures against COVID-19 pandemic worldwide.

## Declarations

### Author contribution statement

Nadim Sharif, Ali Azam Talukder, Shuvra Kanti Dey: Conceived and designed the experiments; Performed the experiments; Analyzed and interpreted the data; Contributed reagents, materials, analysis tools or data; Wrote the paper.

Mithun Kumar Sarkar, Nasir Uddin Nobel, Shamsun Nahar Ahmed, Rabeya Nahar Ferdous: Conceived and designed the experiments; Performed the experiments; Analyzed and interpreted the data; Contributed reagents, materials, analysis tools or data.

Anowar Khasru Parvez: Analyzed and interpreted the data; Contributed reagents, materials, analysis tools or data; Wrote the paper.

### Funding statement

This work was supported by Grants-in-Aid from the 10.13039/501100008804Ministry of Science and Technology, the People's Republic of Bangladesh.

### Data availability statement

Data included in article/supp. material/referenced in article.

### Competing interest statement

The authors declare no conflict of interest.

### Additional information

No additional information is available for this paper.
